# Role of Molecular Biomarkers in Endometriosis-Related Infertility: A Narrative Review of the Literature

**DOI:** 10.7759/cureus.59288

**Published:** 2024-04-29

**Authors:** Nikoleta Koutalia, Fani Gkrozou, Anastasia Vatopoulou, Dimitrios Lentzaris, Chara Skentou, Minas Paschopoulos

**Affiliations:** 1 Obstetrics and Gynaecology, University Hospital of Ioannina, Ioannina, GRC

**Keywords:** pregnancy outcome, subfertility, infertility, biomarkers, endometriosis

## Abstract

Endometriosis is a chronic benign inflammatory disease that affects women of reproductive age. The clinical presentations of endometriosis include dysmenorrhea, dyspareunia, chronic pelvic pain, and infertility. There is a well-established association between endometriosis and infertility. Therefore, there is a need for an early diagnosis of endometriosis-related infertility. In this study, we aim to identify the role of biomarkers as predictive factors of the presence of the disease and its severity and their correlation with the pregnancy outcome.

We performed an electronic database search of all published studies in PubMed and EMBASE from January 2018 to May 2023. Numerous innovative biomarkers identified in cases of endometriosis and infertility have been studied over the past years, including micro-RNAs, BCL6 endometrial expression, cytotoxic T-lymphocyte antigen 4, human leukocyte antigen G, programmed cell death protein 1, programmed cell death ligand 1 immune checkpoint molecules, plasma fibronectin-fibrin complexes, homeobox A10 gene, systemic inflammatory response markers, uterine natural killer cells, and the eutopic endometrium proteome.

Considerable research has been done to identify diagnostic biomarkers for the early detection and prevention of endometriosis-associated infertility. However, none of these biomarkers displayed enough diagnostic accuracy to be used in daily clinical practice. Future research is valuable to establish them as reliable diagnostic tools.

## Introduction and background

Endometriosis is an estrogen-dependent chronic inflammatory disease that affects about 10-15% of reproductive-age women. It is characterized by the presence of endometriotic glands and stroma at extrauterine lesions. The endometrial implants are most commonly located in the pelvic cavity, including the pelvic peritoneum and ovaries, followed by the pouch of Douglas and uterine ligaments. Moreover, endometrial lesions can occur in the intestinal tract or urinary system and, rarely, can affect extrapelvic structures, including the lungs, diaphragm, pericardium, and operative scars. Therefore, endometriosis can appear with various clinical presentations, such as dysmenorrhea, dyspareunia, chronic pelvic pain, and infertility, with 20-25% of patients being asymptomatic [1].

Although the pathophysiology is still under investigation, it is mainly based on Sampson’s theory that endometriosis is caused by retrograde menstruation and the translocation of depleted endometrial cells into the pelvic or abdominal cavity. Even though 90% of women undergo retrograde menstruation, endometriosis occurs in 10% of these cases. It is also proposed that circulating bone marrow-derived stem cells are responsible for the growth and progression of extrauterine endometriotic lesions. A novel theory for the pathogenesis of endometriosis suggests the presence of primitive ectopic endometrial stem cells outside of the uterus cavity during organogenesis. Immune cells, pro-inflammatory cytokines, adhesion molecules, and activated/altered peritoneal microenvironment promote the differentiation, adhesion, and proliferation of these cells. In addition to these theories, genetic predisposition as well as immunological, hormonal, and environmental factors contribute to the development of the disease, as discussed by several studies [2,3].

The classification of endometriosis is most widely based on the revised American Society for Reproductive Medicine (rASRM) staging system. This system evaluates the location, the extent of adhesions, and the depth of infiltration of the endometriotic lesions and classifies endometriosis into stages I to IV. However, the stage does not always correlate with the severity of the symptoms or the response to treatment. The ENZIAN classification system was developed to classify deep infiltrating endometriosis and describe the retroperitoneal structures, but it has poor international acceptance. It is used supplementarily to the rASRM staging system. The endometriosis fertility index is a scoring system developed as a clinical tool to predict postoperative fertility outcomes in women who have undergone surgery for endometriosis. The American Association of Gynecological Laparoscopists classification was developed in 2007 but it is yet to be fully validated and published [4].

To date, laparoscopic inspection of the abdominal cavity and histological confirmation of suspected lesions remains the gold standard method for the definitive diagnosis of endometriosis. The presence of non-specific symptoms as well as the lack of early diagnostic methods can result in approximately 10 years of delay in diagnosis and treatment. Imaging techniques, including ultrasonography and magnetic resonance imaging, are used as a screening or a preoperative tool to determine the exact location of the lesions and the extent of the disease. However, none of these can replace laparoscopy. Consecutively, there is an emerging need to discover more minimally invasive and reliable diagnostic tools with high specificity and sensitivity. The combination of clinical examination, pelvic imaging, genetic tests, and biomarkers could be the key to establishing a less invasive method for an accurate and timely diagnosis of the disease [5].

A biomarker is a characteristic measured and evaluated as an indicator of normal biological processes, pathogenic processes, or pharmacologic responses to an intervention. As mentioned above, endometriosis is a chronic inflammatory process that involves many factors such as hormones, cytokines, glycoproteins, and angiogenic factors. In the past decades, many endometriosis-associated blood biomarkers have been tested, including angiogenic and growth factors, hormonal and inflammatory markers, markers of apoptosis, cell adhesion molecules, other matrix-related proteins, cytoskeleton molecules, nerve growth markers, oxidative stress markers, tumor markers, as well as other peptides and proteins. The development of emerging technologies, such as proteomics, genomics, and mRNA microarrays, may lead to establishing new targets of reliable non-invasive diagnostic biomarkers [6].

Endometriosis can lead to reduced fertility, and it is estimated that 30-50% of women diagnosed with endometriosis experience fertility issues. Bonavina and Taylor suggested that anatomical distortion and the presence of pelvic adhesions may contribute to fertility disorders [7]. Reduced fertility in women with endometriosis can also be related to the altered systemic and peritoneal immune and inflammatory profile. Oxidative stress is linked to infertility as increased production of reactive oxygen species (ROS) leads to the impairment of the endometrial cellular cycle and function [8]. Moreover, it is proposed that chronic intraperitoneal inflammation could lead to decreased folliculogenesis, reduced sperm mobility and sperm-oocyte interaction, embryo transport, and development [7]. The follicular fluid of women with endometriosis consists of altered levels of growth factors and pro-inflammatory cytokines, and this imbalance in the oocyte microenvironment could lead to abnormal oocyte development or result in lower oocyte quality. The diminished ovarian reserve could be explained by the reduction in the amount of healthy tissue due to the presence of endometrioma or due to the presence of increased ROS that affect the normal ovarian cortex. Furthermore, the implantation rate is lower in women with endometriosis. The defective implantation could be due to a reduced endometrial receptivity or decidualization capacity. This can be explained by the chronic pro-inflammatory microenvironment, the hormonal disturbances, and the dysregulation of the genes that are implicated in the implantation process [7,8].

In this study, we aim to identify the role of biomarkers identified in cases of endometriosis and infertility. In the literature, although studies have focused on the recognition of biomarkers in endometriosis, we are attempting to combine their role in women who suffer from endometriosis and infertility at the same time. We aim to recognize their role as predictive factors about the presence of the disease, its severity, and pregnancy outcome.

## Review

Methodology

We performed an electronic database search of all published studies in PubMed and EMBASE from January 2018 to December 2023. The following keywords were used alone or in combination: endometriosis, biomarker, diagnostic, infertility, and fertility disorders. Only scientific papers published in the English language were included. Studies of biomarkers on endometriosis only or infertility only were excluded.

Results

BCL6 Endometrial Expression

The* BCL6* gene encodes a protein that acts as a sequence-specific transcriptional repressor and is involved in the pathogenesis of B-cell lymphoma. The expression of the BCL6 protein leads to the stimulation of pro-inflammatory cytokines (interleukin (IL)-6, IL-8, IL-17) in the peritoneal fluid of these women. Moreover, the overexpression of *BCL6* is related to progesterone resistance in the endometrium, leading to implantation failure and repeated miscarriages. Endometrial *BCL6* forms a complex with *SIRT1*, resulting in the inactivation of progesterone key regulators. Evans-Hoeker et al. confirmed *BCL6* overexpression in the eutopic endometrium of women with endometriosis [9]. Squatrito et al. compared the endometrial expression of *BCL6* between fertile and infertile women with and without endometriosis independently of the stage by using HSCORE analysis [10]. However, HSCORE analysis is known to be biased in its objectivity and reproducibility. It is worth mentioning that this study confirmed that endometrial *BCL6* is significantly overexpressed in women with stage IV endometriosis. Moreover, considering the fertility status, the highest HSCORE was noted in the group of women with infertility compared to women with infertility without endometriosis. Another study concluded that women with suspected endometriosis and elevated *BCL6* expression had lower assisted reproductive technology (ART) success rates compared to those who underwent medical or surgical therapy before embryo transfer [11]. Another study reported that elevated endometrial *BCL6* levels could be used as a positive predictive factor for diagnosing endometriosis in women who undergo in vitro fertilization (IVF). Moreover, Louwen et al. concluded that *BCL6* overexpression could be used as a diagnostic biomarker for patients with endometriosis, and elevated endometrial levels could be used as a negative predictive factor for patients undergoing IVF [12].

Cytotoxic T-lymphocyte Antigen 4, Human Leukocyte Antigen G, Programmed Cell Death Protein 1, and Programmed Cell Death Ligand 1 Immune Checkpoint Molecules

The membrane-bound cytotoxic T-lymphocyte antigen 4 (CTLA4) and programmed cell death protein 1 (PD-1) are immune checkpoint molecules that negatively regulate T-cell activation. Human leukocyte antigen G (HLAG) is a protein that also downregulates the immune system response, and programmed cell death ligand 1 (PD-L1) can combine with PD-1, resulting in the inhibition of the function of activated T cells and cytokine secretion leading to immunosuppression. Santoso et al. studied the presence of these soluble immune checkpoint molecules in the serum and peritoneal fluid of women with endometriosis-related infertility. The following two groups were involved: the endometriosis group and the control group which included non-endometriosis women who had fibroids or other single benign gynecological pathology of the fallopian tubes or ovaries. Increased serum levels of sPD-L1 were detected in patients with endometriosis-related infertility, and more precisely the levels were nearly twice higher compared to control (19.33 pg/mL, 14.47-49.77 vs. 10.45 pg/mL, 5.48-14.40; p < 0.0001). On the other hand, there was no difference in the serum levels of sCTLA4, sHLAG, and sPD-1 compared to the control group. Moreover, patients with late-stage endometriosis had significantly higher serum levels of sCTLA-4 and sPD-L1 compared to early-stage endometriosis. Considering the peritoneal fluid, all immune checkpoint molecules were increased in endometriosis-related infertility cases [13]. This study highlighted that serum levels of sPD-L1 can be used as a candidate biomarker in endometriosis-related infertility and late-stage endometriosis. Although the concentration of peritoneal sPD-1 and sHLAG has good diagnostic accuracy, it is less likely to be used widely as less invasive biomarkers are preferred in clinical settings. Abramiuk et al. studied the expression of CTLA4 on the surface of T and B lymphocytes and determined the concentration of sCTLA4 antigen in peripheral blood plasma and peritoneal fluid in patients with endometriosis-related infertility. CD4+ and CD8+ T lymphocytes were negatively correlated with the percentage of natural killer (NK) and NKT-like cells, while a positive correlation between CD8+ T cells and the percentage of Treg CD4+CD25+highFoxp3 was reported. Furthermore, significantly higher percentages of CD4+ CTLA4 and CD8+ CTLA4 T cells were detected in endometriosis patients with intraoperative adhesions [14]. These studies concluded that CTLA4-linked autoimmunity is involved in the pathogenesis of endometriosis and related infertility. However, further investigation on their role in diagnosis and targeted immunotherapy is needed.

Plasma Fibronectin-Fibrin Complexes

Plasma fibronectin (FN)-fibrin complexes support platelet aggregation, stabilize thrombus growth, and play an important role in ensuring the balance between hemorrhage and excessive blood clot formation which causes blood occlusion. Lis-Kuberka et al. studied the presence of plasma FN-fibrin complexes in women with endometriosis and fertility disorders. Blood samples from women with endometriosis (n = 38), fertility disorders (n = 28), and healthy women (n = 25) were collected. FN-fibrin complexes of high molecular mass were detected in the plasma of women with infertility and patients with endometriosis. On the contrary, macromolecular FN-fibrin complexes (more than 1,300 kDa) were not detected in healthy women. More specifically, FN-fibrin complexes with molecular masses of 750, 1,000, 1,300, 1,600, and 1,900 kDa were found in both groups of endometriosis and fertility disorders, and constituted 50.55% and 56.57% of all molecular forms, respectively, whereas in the healthy group, constituted only 13.24% of all forms. The FN-fibrin complex of 1,900 kDa was detected in two out of 38 plasma samples of the endometriosis group and four out of the 28 samples of the fertility disorders group; however, it was not detected in the normal group [15]. The study suggested that fertility disorders (regardless of the presence of endometriosis) are related to an increased and chronic activation of the coagulation cascade. The presence of these high molecular complexes could be used as a potential non-invasive biomarker for the early detection of fertility disorders and endometriosis. However, some limitations may have an impact on the findings of the study. First, women in the control group were younger compared to those with endometriosis and fertility disorders. Moreover, the exclusion criteria for women with endometriosis and fertility disorders did not include hormonal disorders, polycystic ovary syndrome, and metabolic disorders, such as obesity and insulin resistance, leading to questioning the influence of these disorders on the formation of FN-fibrin complexes.

Homeobox A10 Gene

Homeobox A10 (*HOXA-10*) is a protein-coding gene expressed in the endometrium in a cyclical manner. Steroid hormones, estrogen, and progesterone stimulate endometrial expression, which is upregulated during the implantation window, especially during the intermediate secretory phase of the menstrual cycle. Özcan et al. conducted a prospective clinical study to compare *HOXA-10* gene expression in eutopic endometrium samples between women who were fertile with endometriosis and women with endometriosis-related infertility and control fertile cases without endometriosis. Furthermore, this study aimed to compare *HOXA-10* gene expression in endometrial samples and endometrioma specimens with the severity of the disease. A total of 33 patients, who were in the secretory phase after ovulation, were included and divided into three groups. The first group consisted of 11 fertile control cases without endometriosis, whereas both the second and the third group consisted of 11 fertile and 11 infertile women with endometrioma, respectively. This study concluded that *HOXA-10* gene expression in the eutopic endometrium samples of women with endometriosis is significantly downregulated compared to the eutopic endometrium samples of control cases. More precisely, *HOXA-10* gene expression was 3.5-fold downregulated in women with endometriosis-related infertility compared to those without endometriosis. Moreover, this study suggests that patients with endometriosis and infertility have lower levels of endometrial *HOXA-10* gene expression than those without fertility disorders. Conclusively, severe endometriosis cases express significantly lower levels of the gene in both the endometrium and endometrioma tissues compared to moderate endometriosis cases. However, it is worth mentioning some of the study limitations. For instance, the inclusion criteria for the first group were based on ultrasonography, and the absence of endometriotic lesions was not visually confirmed by laparoscopy. Another limitation was the lack of endometrial sample dating that predicts the implantation window period. Furthermore, the study did not evaluate the expression of HOXA-10 encoded protein in endometrial samples and did not provide information about gene expression and fertility outcomes after the surgery [16].

Micro-RNAs

Micro-RNAs (miRNAs) are single-stranded, highly conserved, small non-coding RNAs (21-22 nucleotides long) that play an important role in post-transcriptional gene regulation. They bind to the 3΄-untranslated regions (3΄-UTR) of their targeted mRNAs leading to translational repression and they have an impact on mRNA stability/degradation. miRNAs are present intracellularly and in biological fluids (plasma, serum, peritoneal fluid) with high stability. The altered miRNA expression profile pattern is associated with many human diseases, including benign and malignant disorders of the human female reproductive system. Dysregulation of mi-RNA plays an important role in the pathogenesis of endometriosis through moderating inflammation, angiogenesis, proliferation, and tissue remodeling. In the last decade, many studies have been conducted to identify the miRNAs with differential expression in endometriosis, the factors that influence miRNA expression, as well as their role as a candidate biomarker in patients with endometriosis [17]. miRNA studies have helped reveal the molecular mechanisms that associate endometriosis with infertility. *HOXA-10*, aromatase, progesterone receptors, matrix metalloproteinases (MMPs), and alphaV beta 3-integrin are some of the genes with altered expression in eutopic endometrium in women with endometriosis and fertility disorders. As mentioned, *HOXA-10* is upregulated in the endometrium during the implantation window in healthy women. Studies have shown that miR-135a/b is upregulated in the proliferative phase in patients with endometriosis-related infertility causing repression of the transcription factor HOXA-10. Moreover, *lncRNA H19*, which is normally expressed in the eutopic endometrium, binds to let-7miRNA leading to a reduction of its bioavailability. A previous study proposed that H19 expression reduces in patients with endometriosis, leading to an increased let-7 activity that consecutively inhibits *IGF1R* expression, resulting in decreased stroma cell proliferation and impaired endometrial receptivity. On the contrary, Liu et al. concluded that *lncRNA H19* overexpression in the ectopic and eutopic endometrium was positively correlated with infertility. In both studies, endometrial tissue samples were collected during the late proliferative phase of the menstrual cycle [17,18].

Progesterone resistance is also associated with endometriosis and infertility and is considered a crucial factor for decreased endometrial receptivity and implantation failure. Human endometriosis patients and baboons with induced endometriosis have been studied, demonstrating that miR-29c expression levels were increased while the targeted transcript levels of FK506-binding protein 4 (FKBP4), a progestin receptor co-chaperone, were decreased. In addition, surgical resection of the endometriotic lesions is likely to reverse these expression changes. This study was limited by its low sample size (nine endometrial biopsy specimens preoperatively), especially postoperatively, as only four endometrial biopsy samples were studied [19]. Furthermore, another study suggested that miR-194-3p is overexpressed in the eutopic endometrium in the initial stages of endometriosis, resulting in a significant reduction of both PR-A and PR-B expression levels. Consecutively, miR-194-3p overexpression is associated with progesterone resistance and impaired decidualization in endometrial stroma cells [20]. A previous study reported that both miR-196a and MEK/ERK signaling are upregulated in the eutopic endometrium of women with minimal or mild endometriosis leading to the downregulation of PGR expression. Another study investigated the role of miR-125b and its target gene, MMP26, and their association with endometrial receptivity in women undergoing IVF-embryo transfer. The study clarified that miR-125b was significantly upregulated in endometrial epithelial cells (EECs) in women with elevated progesterone on the day of human chorionic gonadotropin administration [17]. miR-125b can inhibit MMP26 protein translation, and the decreased levels of MMP26 consecutively affect EEC migration, decreasing the invasion capacity and, therefore, the implantation sites.

Marí-Alexandre et al. described, for the first time, the miRNA profile in the peritoneal fluid of women with endometriosis and the association of miRNA levels with fertility status. In general, 126 miRNAs were differentially expressed (78 downregulated and 48 upregulated) in the peritoneal fluid of patients with endometriosis. Significantly higher levels of miR-106b-3p, -451a, and -486-5p were reported in the peritoneal fluid of sterile women with endometriosis, suggesting their potential role in predicting the fertility outcome of these women [21]. A more recent study reported a saliva-based miRNA signature profile as an innovative diagnostic screening tool for endometriosis-related infertility [22].

Systemic Inflammatory Response Markers

Endometriosis is associated with chronic intraperitoneal inflammation, including increased inflammatory cytokines, chemokines, prostaglandins, white blood cells, and, especially, macrophages, creating an immunotolerant microenvironment that allows endometriotic implants to survive. Endometrial implants secrete transforming growth factor-β (TGF-β), vascular endothelial growth factor (VEGF), IL-1, IL-6, IL-8, tumor necrosis factor-alpha (TNF-α), and other proinflammatory cytokines. This inflammatory and angiogenic intraperitoneal microenvironment reduces the motility of sperm, impairs the oocyte and embryo quality, and leads to implantation dysfunction. Freitag et al. studied the eutopic endometrial immunological changes of women with endometriosis-related infertility and observed elevated levels of macrophages, NK cells, or plasma cells, as well as higher levels of endothelial VEGF-A expression [23]. It has been reported that women with endometriosis have increased neutrophils and decreased lymphocytes. Jing et al. concluded that serum neutrophil-to-lymphocyte-ratio (NLR) and the combination of NLR and cancer antigen 125 (CA-125) in patients with endometriosis-related infertility was significantly lower than in fertile patients [24]. Therefore, serum NLR can be used as a potential predictive marker for endometriosis with infertility. The limitation of this study was that healthy women were not included. Kolanska et al. studied serum, peritoneal, and follicular fluid cytokines, immune markers, and autoantibodies as well as their role in endometriosis-related infertility [25]. Follicular fluid levels of TNF-α, granulocyte-macrophage colony-stimulating factor, and IL-5 were increased in this group of patients whereas IL-10 appeared to be decreased. TNF-α concentration in the follicular fluid was correlated with impaired oocyte quality. Although IL-6 levels were not significantly increased in the follicular fluid, endometrial stroma cell implants secreted increased levels of IL-6 that could be due to local production or after IL-1 stimulation. Of note, IL-1β is elevated in endometriosis tissues. IL-1β inhibits estrogen receptor a and progesterone receptors A and B, impacting endometrial differentiation and decidualization. On the other hand, increased levels of IL-6 could inhibit endometrial human stroma cell proliferation, reduce sperm motility, and may limit the rate of blastocyst attachment with matrix substrates. Considering the peritoneal fluid, significantly higher levels of TNF-α, IL-6, and IL-8 were detected in comparison with fertile women. Lin et al. reported that patients with endometriosis-related infertility had significantly higher volumes of peritoneal fluid. Furthermore, the study suggested that women with endometriosis-related infertility presented increased levels of TNF-α, IL-1, IL-6, IL-10, TGF-β1, and IL-8 in peritoneal fluids [26]. Considering the follicular fluid of patients with endometriosis, another study reported that high levels of IL-8 and IL-12 in patients undergoing IVF are associated with impaired oocyte maturity and embryo quality. A precious study investigated the peritoneal cytokine profile of women with infertility and discovered that 11 cytokines were differently expressed in patients with endometriosis [26]. Five cytokines were negatively correlated with endometriosis (IL-13, IL-5, cutaneous T-cell-attracting chemokine, monocyte chemotactic protein (MCP)-3, and macrophage colony-stimulating factor), and six were positively correlated (stem cell growth factor-beta, hepatocyte growth factor, interferon α2, MCP-1, IL-9, and leukemia inhibitory factor). Unfortunately, the 11 class-separating cytokines had a misclassification rate of 30-40%, which immediately decreased their importance as predictive markers [26].

CD4+ T lymphocytes, or Th cells, can be further subdivided into Th1 and Th2 cells. The cytokines that are produced are known as Th1-type, which elicit pro-inflammatory responses, and Th2-type, which generate anti-inflammatory responses. As mentioned earlier, a shift toward Th2 response is observed in endometriotic lesions. The reduced Th1/Th2 ratio in the peritoneal fluid could be associated with endometriosis-related infertility [26].

Wang et al. found that patients with endometriosis-related infertility had higher levels of IL-6, IL-10, IL-13, and TNF-α in peritoneal fluid compared with those in the control group [27]. Two prospective, non-randomized studies were conducted by Gica et al. to evaluate the use of biomarkers in predicting infertility in women with non-obstructive endometriosis. In the first study, CA-125 was tested in 116 women, and in the second, IL-6 and IL-8 were tested in 36 women. The study showed that increased serum levels of IL-6 and CA-125 could be used as predictive biomarkers for infertility in women with non-obstructive endometriosis. However, the limitation of the study was the difference in the sample size of each study. Moreover, the second study suggested that fertility status was significantly correlated with the stage of endometriosis, whereas the first study suggested that there was no significant difference [28].

Uterine Natural Killer Cells

In 2022, Shih et al. proposed the single-cell analysis of menstrual endometrial tissues. The study included the following three groups: the control group (n = 9), the endometriosis group (n = 11), and the symptomatic subjects (n = 13) who had not been diagnosed with endometriosis yet. Endometrial tissue fragments derived from menstrual effluent (ME) were collected and single-cell RNA sequencing (scRNA-Seq) analysis was performed. A remarkable reduction of total uterine NK (uNK) cells in the ME tissues of endometriosis cases was demonstrated (approximately 8% in cases and 28% in healthy controls). Furthermore, fractions of both uNK1 and uNK2 subclusters were significantly increased in the ME tissues of controls compared to endometriosis cases. More specifically, the uNK2 subcluster, which is characterized by the expression of genes associated with cell proliferation, was enriched in controls and depleted in endometriosis cases [29]. uNK cells are most prominent in the first trimester of pregnancy, as they regulate trophoblast invasion and spiral artery remodeling. This indicates that uNK cells play a role in placental implantation. Moreover, uNK cells contribute to senescent decidual cell removal, and a lack of them is associated with increased senescent cells in the stromal subclusters. The findings of this study are in contrast with Freitag et al. [23] and other studies which indicated that endometriosis patients are more prone to elevated uNK cells. Cytotoxic uNK cell surface receptors, CD16+ and NKp46+, are significantly increased in women with endometriosis, suggesting that these uNK cells may have higher levels of cytotoxic activity. As uNK cells are reported to play a role in successful implantation, defects in uNK cell phenotype and function may contribute to endometriosis-related infertility. Further analysis regarding this will be of interest.

Proteomic Profile of Eutopic Endometrium

The study of proteomic technologies has expanded in recent years opening up new possibilities in discovering potential diagnostic biomarkers. Proteins are differentially expressed during the proliferative stage of the menstrual cycle in the eutopic endometrium of women with endometriosis. Yao et al. studied the proteomic changes of eutopic endometrium in patients who suffered from endometriosis-related infertility [30]. In their study, 6,698 proteins were identified and 5,812 proteins were quantified. It was reported that 16 proteins were upregulated and 23 proteins were downregulated in the eutopic endometrium of women with endometriosis-related infertility when compared with the healthy control group. The upregulated proteins were related to the acute-phase response, the immunoglobulin-mediated immune response, the antimicrobial humoral response, the regulation of the humoral immune response, the B-cell-mediated immunity, the regulation of the inflammatory response, and the regulation of the protein activation cascade. On the other hand, the proteins that were downregulated were related to the positive regulation of interferon-gamma production, the regulation of the necroptotic process, the regulation of necrotic cell death, T-cell activation, the regulation of leucocyte proliferation, and the positive regulation of hemopoiesis. Furthermore, alpha-1-acid glycoprotein 2, an acute-phase response protein, complement factor B, a component of the alternative pathway of complement, zinc transporter Zip14, which responds to pro-inflammatory stimuli, and many other proteins were significantly upregulated. These proteins are associated with the humoral immune pathway and the acute-phase response, confirming the previous theories and studies that endometriosis-related infertility is linked with systemic inflammation and an impaired immune system.

Table 1 presents a summary of candidate biomarkers associated with endometriosis-related infertility.

**Table 1:** Summary of candidate biomarkers associated with endometriosis-related infertility. CTLA4 = cytotoxic T-lymphocyte antigen 4; HLAG = human leukocyte antigen G; PD-1 = programmed cell death protein 1; PD-L1 = programmed cell death ligand 1; FN = fibronectin; HOXA-10 = homeobox A10; miRNA = microRNA; NLR = neutrophil-to-lymphocyte ratio; uNK = uterine natural killer

Candidate biomarkers	Cases with endometriosis-related infertility
BCL6 endometrial expression	Endometrial BCL6 overexpression
CTLA4, HLAG, PD-1, PD-L1 immune checkpoint molecules	Increased serum levels of sPD-L1
	Increased levels of all four molecules in the peritoneal fluid
	Good diagnostic accuracy of peritoneal sPD-1 and sHLAG
	CTLA4-linked autoimmunity is involved in the pathogenesis of endometriosis-related infertility
Plasma FN-fibrin complexes	Detection of macromolecular plasma FN-fibrin complexes
HOXA-10 gene	Downregulation of HOXA-10 gene expression in eutopic endometrium
miRNAs	Upregulation of miR-135a/b in the proliferative phase causing repression of the transcription factor HOXA-10
	Inconclusive results considering the lncRNA H19 endometrial expression
	Progesterone resistance is associated with the overexpression of miR-29c and miR-194-3p and the upregulation of miR-196a and MEK/ERK signaling in the eutopic endometrium
	Increased levels of miR-106b-3p, -451a, and -486-5p in the peritoneal fluid
	Innovative research on saliva-based miRNA signature profile
	Available data cannot conclude a more frequent or important panel of miRNAs
Systemic inflammatory response markers	Lower serum NLR than fertile endometriosis cases
	Greater peritoneal fluid volume
	Reduced Th1/Th2 ratio in the peritoneal fluid
	Available data cannot conclude a more frequent or important cytokine profile in serum, follicular, and peritoneal fluid
uNK cells	Defective uNK cell phenotype and function
Proteomic profile of eutopic endometrium	Upregulation of alpha-1-acid glycoprotein 2, complement factor B, zinc transporter Zip14, and many other proteins associated with the acute-phase response, the immunoglobulin-mediated immune response, the antimicrobial humoral response, the regulation of humoral immune response, B-cell-mediated immunity, the regulation of inflammatory response, and the regulation of protein activation cascade

## Conclusions

Endometriosis is a chronic disease seen in many reproductive-age women all over the world. It is characterized as a chronic, inflammatory, estrogen-dependent disease, the pathogenesis of which is multifactorial. Moreover, no theory can be associated with all of the described comorbidities of endometriosis. Endometriosis-related infertility has been extensively researched over the years. The mechanisms that have been proposed to associate endometriosis with infertility include chronic pain and inflammation, pelvic adhesions and anatomy distortion, ovarian dysfunction, and defective endometrial receptivity. Over the decade, considerable research has been done to identify diagnostic non-invasive biomarkers that could potentially contribute to the prediction of a higher risk of infertility in women who suffer from endometriosis. So far, none of these biomarkers are used in clinical practice but further research needs to be made to establish them as diagnostic tools for the early detection and prevention of endometriosis-associated infertility. Inflammatory cytokines are not suitable to diagnose patients with endometriosis. A biomarker panel that combines several different markers will most likely be more accurate in diagnosing endometriosis related to fertility than any single biomarker.

This review focuses on the innovative possible biomarkers expressed in serum, plasma, peritoneal fluid, eutopic, and ectopic endometrium of women with endometriosis-related infertility. However, most studies suffer from limitations that include invasive methods of biomarker sampling, inclusion criteria based only on ultrasonography, a limited number of patients, the presence of other hormonal or metabolic disorders, the lack of analyzing gene expression or fertility outcomes after therapy or surgery, and other numerous setbacks. In addition, the number of meta-analyses about diagnostic markers is limited. Research on biomarkers is open and valuable. Future new methods may help solve this problem of early diagnosis of endometriosis mainly for women who deal with fertility issues.
